# Self-Perceived Stress During the Quarantine of COVID-19 Pandemic in Paraguay: An Exploratory Survey

**DOI:** 10.3389/fpsyt.2020.558691

**Published:** 2020-10-26

**Authors:** Julio Torales, Carlos Ríos-González, Iván Barrios, Marcelo O'Higgins, Israel González, Oscar García, João Mauricio Castaldelli-Maia, Antonio Ventriglio

**Affiliations:** ^1^Department of Psychiatry, School of Medical Sciences, National University of Asunción, San Lorenzo, Paraguay; ^2^Research Department, School of Medical Sciences, National University of Caaguazú, Coronel Oviedo, Paraguay; ^3^Department of Neuroscience, Medical School, Fundação do ABC, Santo André, Brazil; ^4^Department of Psychiatry, Medical School, University of São Paulo, São Paulo, Brazil; ^5^Department of Clinical and Experimental Medicine, University of Foggia, Foggia, Italy

**Keywords:** COVID-19, pandemic, stress, perceived-stress, Paraguay, mental health

## Abstract

**Introduction:** Any viral pandemic is a global health and mental health issue. The World Health Organization and mental health associations have warned that the current COVID-19 pandemic will lead to a drastic increase of stress-related conditions and mental health issues globally.

**Materials and Methods:** An online web-based survey has been launched from 10 to 15 April 2020 in Paraguay in order to collect information regarding the stress related to the quarantine during the COVID-19 pandemic. It has been spread through social media (“*WhatsApp*,” “*Twitter*,” and “*Facebook*”). Two thousand two hundred and six Paraguayan citizens, over 18 years of age, completed the survey voluntarily. Socio-demographics as well as ratings at Self-perceived Stress Scale have been collected and analyzed.

**Results:** Two thousand two hundred and six subjects (74.12% men) aged between 18 and 75 with an average of 34 ± 11 years old completed the survey. 12.42% (276 subjects) of sample reported a preexisting diagnosis of mental disorder, and 175 participants (7.93%) reported an increase of preexisting symptoms with the onset of COVID-19 quarantine. 41.97% of them had anxiety and 54.38% did not receive any specific treatment. The general population rated 18.10 ± 5.99 at Self-perceived Stress Scale, which indicates a moderate level of self-perceived stress. Significant association was found between higher levels of stress and female sex, being single, or reporting preexisting mental disorder, above all anxiety and depression (*p* < 0.01). In fact, in 63.87% of mentally ill subjects (*n* = 175), the quarantine has worsened symptoms of preexisting mental disorders.

**Conclusion:** This study suggests a stressful impact of COVID-19 pandemic, with the majority of participants reporting a moderate level of self-perceived stress. We suggest mental health services to provide a phone-based or web-based support to the general population in order to contrast the psychological impact of the pandemic. This approach may improve the accessibility to mental healthcare services in Paraguay, especially in times of social distancing.

## Introduction

Any viral pandemic is a global health and mental health issue (1). The impact of a pandemic outbreak on the global health relies on the characteristics of the virus, evidence of rapid human-to-human transmission, the severity of the resulting disease, and the medical and non-medical resources available to control the impact of the virus (e.g., vaccines, treatment drugs, isolation protocols, economic resources to support the lockdown, etc.) (2). The Coronavirus SARS-CoV-2, causing the COVID-19 disease, has spread rapidly through several countries worldwide leading to a pandemic in March 2020 as recognized by the World Health Organization (WHO) (3, 4). In Paraguay, cases of infection have increased sharply in a few days as in the rest of the world (5). It has been argued that feelings of fear, uncertainty, loneliness as well as stress, anxiety, and depression have been reported in the general population worldwide after the outbreak of COVID-19 (6). Also, four potential stages of psychological response to the pandemic have been proposed: stress and fear, anxiety and panic, anger and denial, acceptance, and resolution (6–8). Also, isolation, quarantine and social distancing had a relevant impact on the subjective well-being and level of personal stress (8).

The World Health Organization as well as the international mental health associations have warned that the current COVID-19 pandemic will lead to a drastic increase of stress-related conditions and mental health issues globally (9). In fact, emerging reports have been documenting an increase of stress-related symptoms, anxiety and depression especially among vulnerable populations such as socially and economically disadvantaged people, chronically, and mentally ill populations (10, 11). Also, this condition may add severity to negative prognoses of physical as well as mental diseases with adjunctive difficulty in accessing health services (9, 12). For these reasons, psychological support and crisis interventions should be promoted to contrast the effects of the pandemic (10). Psychological support should be offered to vulnerable subjects, healthcare workers as well as to general population. Moreover, advices to the general population on how to cope with subjective stress during the pandemic may be helpful (10). Measures of stress in the epidemiological research may include three components: (a) *environmental*, including stressful life *events*; (b) *psychological*, which involves subjective experience and emotional response to stressors; and, (c) *biomedical*, which comprises the physiological systems involved in coping the stressful stimuli (13).

This survey aimed to investigate the level of stress perceived by the general population during the current COVID-19 global pandemic and quarantine in Paraguay (14). We expected to reveal a significant self-reported subjective stress on a large scale, above all among people reporting preexisting mental health problems. We employed The Perceived Stress Scale (PSS), a well-known and validated tool, originally developed in 1983, which scores the level of subjective perceived stress (e.g., feelings and thoughts; *psychological* component) related to conditions and stressful life events occurred in the last month (*environmental component*) Also, factors associated to higher perceived stress have been described.

## Materials and Methods

This was an observational, cross-sectional study, based on an online survey launched from 10 to 15 April 2020, one month after the implementation of preventive actions (quarantine) according to the Decree 3442/20 of the Presidency of the Republic of Paraguay (15). Two thousand two hundred and six Paraguayan citizens, of both sexes, over 18 years of age, voluntarily completed the survey, nationally spread through social media (“*WhatsApp*,” “*Twitter*,” and “*Facebook*”). All participants received complete information about the aim of the study, privacy and data—processing. No payment has been foreseen for completing the survey.

The Spanish version of the Perceived Stress Scale-10 (EEP-10), as used by Remor in a validation-study with adults in Spain (16), has been employed in this study. This rating scale measures the perception of psychological stress during the last month, and reports on situations of daily life which are considered as stressful (specified as the outbreak of COVID-19 and quarantine in this survey). Also, it explores the level of stress experienced over the last month on a 5-point scale (0–never, 1 = almost never, 2 = once in a while, 3 = often, 4 = very often). Six of the 10 items are worded and scored in the non-reversed direction (i.e., “how often have you felt that you were unable to control the important things in your life”). Four of the 10 items are worded and scored in the reversed direction (i.e., “how often have you felt that things were going your way”) (17). The scale is easily understandable by the general population, independently of education level. Also, this scale shows an adequate reliability (α = 0.82, test-retest, *r* = 0.77), validity, and sensitivity (16).

The scale total score ranges from 0 to 40. Scores ranging from 0–13 would be considered as low self-perceived stress. Scores ranging from 14–26 would be considered as moderate self-perceived stress. Finally, scores ranging from 27–40 would be considered as high self-perceived stress (17–19).

The study was approved by the Ethics Committee of the School of Medical Sciences at the National University of Caaguazú, Paraguay. Data were treated with confidentiality, equality, and justice, respecting the Helsinki principles. Participants who required a feedback from the questionnaire were invited to write down their email address and received information or specific helpful suggestions.

Data were stored in a Microsoft Office Excel 2013 file and processed with RStudio statistical package version 1.2.5033 for analysis. The results are expressed in tables of proportions. Associations were tested with Student's T distribution and ANOVA, as appropriate. Statistical significance was considered for *p* ≤ 0.05.

## Results

Two thousand two hundred and six participants completed the web-based survey, 74.12% (*n* = 1,635) were men, aged from 18 to 75 with an average of 34 ± 11 years old. 49.77% (*n* = 1,098) were single and 77.24% (*n* = 1,704) reported an university education. 36.18% (*n* = 935) reported an independent work or working for a private enterprise. Socio-demographic characteristics of the sample are shown in Table 1.

**Table 1:** Socio-demographic characteristics of the sample (*N* = 2,206).

**Characteristic**	***n***	**Frequency (%)**
**Sex**		
Man	1,635	74,12
Woman	563	25,52
I'd rather not say it,	8	0,36
**Marital Status**		
Single	1,098	49,77
Married	688	31,19
Stable union	258	11,70
Separate	81	3,67
Divorced	68	3,08
Widow	13	0,59
**Education**		
None	28	1,27
Primary	27	1,22
Secondary	231	10,47
University	1,704	77,24
Tertiary (non-university)	216	9,79
**Urban area**		
Asunción (capital city)	740	33,54
Central (surroundings of the capital)	653	29,60
Other cities	813	36,86
**Employment**		
Public sector	711	27,52
Independent/private	935	36,18
Unemployed	348	13,47
Undergraduate student	463	17,92
Graduate student	127	4,91

Participants were invited to report on their mental health, providing an answer to the following questions: *Are you diagnosed with a mental disorder? If the answer is yes, could you indicate which one? Are you receiving any type of treatment? If the answer is yes, could you indicate what type?* 12.42% (*n* = 274) reported a preexisting diagnosis of mental disorder and 63.87% of them (*n* = 175) considered that their symptoms have worsened with the start of the quarantine. 41.97% (*n* = 115/274) of them reported anxiety and 54.38% (*n* = 149) were not under any specific treatment. The information about the mental health of the participants is summarized in Table 2.

**Table 2:** Mental health of participants: subjects reporting preexisting mental disorders (*n* = 274).

**Characteristics**	***n***	**Frequency (%)**
**Diagnosis**		
Generalized anxiety disorder	115	41,97
Anxiety and depression	65	23,72
Major depressive disorder	48	17,52
Panic disorder	24	8,76
Borderline personality disorder	10	3,65
Obsessive-compulsive disorder	4	1,46
Post-traumatic stress disorder	2	0,73
Bipolar disorder	2	0,73
Schizophrenia and other psychotic disorders	2	0,73
Impulse control disorder	1	0,36
Attention deficit and hyperactivity disorder	1	0,36
**Treatment**		
No, I am not receiving any treatment	149	54,38
Yes, psychological	37	13,50
Yes, psychotropics	52	18,98
Yes, psychotropics, and psychological	36	13,14

Regarding the Self-Perceived Stress Scale, the internal consistency of ratings with the Cronbach alpha was 0.84. The mean score of the population was 18.10 ± 5.99 (moderate stress level). 67.49% of people without mental illness also reported a moderate level of self-perceived stress, as well 71.17% of people with mental illness (*p* < 0.001). Reported stress levels are detailed in Table 3.

**Table 3:** Self-Perceived stress levels in the sample.

**Self-Perceived stress level**	**Without mental illness (*****n=*** **1,932)**		**With mental illness (*****n=*** **274)**		**Total sample (*****N=*** **2,206)**	
	***n***	**Frequency (%)**	***n***	**Frequency (%)**	***n***	**Frequency (%)**
Low stress	473	24.48	17	6.20	490	22,21
Moderate stress	1,304	67.49	195	71.17	1,499	67,95
High stress	155	8.02	62	22.63	217	9,84

The mean score of self-perceived stress among men was 16.82 ± 5.52 (moderate level) whereas women scored 18.54 ± 6.07 (also considered as a moderate level of self-perceived stress). Significant associations were found between stress scores and sex, marital status, reported preexisting mental disorders, and age (Table 4): women perceived more stress than men during the quarantine as well as single subjects and those self-reporting a preexisting mental illness (the size of the effect was based on a Cohen's d equal to 0.71). Lower age was significantly associated with higher SPS scores. In the bivariate comparison, the male-female pair was statistically significant with the Turkey test (*p* < 0.0001). Regarding marital status, all pairs were significant (*p* < 0.05) except for the married-widowed pair.

**Table 4:** Factors associated to self-perceived stress in the sample (*N* = 2,206).

**Factors**	**Self- Perceived stress**		***p-*value**
	**Mean** **±** **Standard deviation**		
**Sex**			*p < * 0.0001
Women	18,53	5,52	
Men	16,81	6,07	
I would rather not say	19,12	7,60	
**Marital Status**			*p < * 0.0001
Single	19,38	6,06	
Married	16,08	5,23	
Stable union	17,84	6,07	
Separate	18,14	6,07	
Divorced	18,67	6,15	
Widow	17,84	4,02	
**Education**			0.126
None	19,53	6,03	
Primary	19,33	4,28	
Secondary	18,77	6,06	
Tertiary (non-university)	17,66	6,10	
University	18,02	5,98	
**Urban Area**			0.446
Asuncion (capital city)	18,01	5,89	
Central (surroundings of the capital)	18,34	6,18	
Other cities	17,98	5,91	
**Psychiatric diagnosis**			*p < * 0.0001
Yes	21,74	5,78	
No	17,58	5,83	
**Age**			*p < * 0.001

Mental health factors associated to higher levels of self-perceived stress included any preexisting diagnosis (Table 5), with a greater significant association for preexisting anxiety and depression (*p* < 0.01). In addition, 175 participants (7.93% of the whole sample or 63.87% of 274 mentally ill subjects) reported a subjective worsening of symptoms with the onset of COVID-19 quarantine.

**Table 5:** Mental health factors associated to Self-perceived stress in mentally ill subjects (*n* = 274).

**Factors**	**Self- Perceived stress**		***p-*value**
	**Mean** **±** **Standard deviation**		
**Diagnosis**			0.00679
Generalized anxiety disorder	20,72	5,73	
Anxiety and depression	24,01	5,03	
Major depressive disorder	21,52	5,81	
Panic disorder	22,45	6,32	
Borderline personality disorder	23	4,13	
Obsessive-compulsive disorder	17	3,83	
Post-traumatic stress disorder	23	14,14	
Bipolar disorder	16	1,41	
Schizophrenia and other psychotic disorders	13,5	7,77	
Impulse control disorder	18		
Attention deficit and hyperactivity disorder	21		
**Treatment**			0.336
No, I am not receiving any treatment	21,49	5,44	
Yes, psychological	23,19	6,06	
Yes, psychotropic	21,13	5,92	
Yes, psychotropic, and psychological	22,19	6,59	

## Discussion

Most of mentally ill subjects (63.87%) reported that COVID-19 quarantine has been worsening symptoms of preexisting mental disorders. As expected and warned by WHO (9), isolation and restrictions may exacerbate feelings of anxiety, fear and anger, especially among subjects suffering from preexisting mental distress (6, 20, 21). They also may be at higher risk of developing additional symptoms of post-traumatic stress disorder (20). Patients suffering from mental health disorders, because of the adjunctive perceived stress during the quarantine, need specific interventions in order to reduce the negative impact of infection and isolation on their own clinical outcome and quality of life (22). In addition, in our sample, there were no significant different levels of perceived stress among subjects with preexisting mental illness receiving or not- receiving any treatment (psychological or pharmacological).

Emerging symptoms among people without preexisting conditions, after the exposure to collective stressful events, such as depressive symptoms, irritability, insomnia, anxiety as well as functional neurological symptoms have been reported (23, 24). Our study confirmed significant levels of stress, even if different, among subjects with and without mental health conditions. Also, in the sample, greater stress has been perceived as associated to anxious and depressive symptoms, according to some previous studies on quarantine (25).

In 2015, during a quarantine in Korea and Ebola viral epidemic in Sierra Leone, greater stress was associated with the female sex, university education level (25, 26). In our survey, the association with female sex has been confirmed whereas no significant association was found with the education level: this may be due to a selection bias since most of participants (1,704; 77.24%) reported to be graduated. This may suggest that people (77.24% of sample) with higher level of education, as well as more accessibility to internet, completed the survey more easily than those with lower level of education.

The internal consistency of ratings found in this study was greater than those found in studies conducted in Colombia or Korea with a Cronbach alpha value of 0.65 and 0.70, respectively (27, 28). In fact, the internal consistency value (0.84) is relevant especially for a complex construct (such as perceived stress) measured in large heterogeneous sample (2,206 subjects from the general population) (29).

This survey was designed to measure the short term (10–15 April 2020) perceived stress following the first wave of COVID-19 and lockdown in the general population in Paraguay, it does not aim to measure the perceived stress among subjects with mental disorders specifically: the instrument used for the survey is adequate for this purpose. The survey was launched through some social networks and apps, as this allowed to reach a large part of general population in a short period of time, considering that in-person assessment would have been impossible due to the lockdown and social distancing measures imposed in the country. Questions regarding preexisting conditions were included in order to determine whether their presence could affect the perceived stress in the general population. Further research will be needed to determine the perceived stress in patients with mental disorders.

Limitations of the survey may include the absence of specific data on psychological or pharmacological interventions for subjects under treatment, as well as the lack of a comparison between the subjects with mental disorders and the general population: our numbers did not allow for any matched comparison. Another limitation of the design is the difficulty to separate the impact of the quarantine and the fear of the virus itself, there may be different factors that could be better measured with different designs. The use of social media to distribute the screening tool could negatively impact the ability to reach people with mental illness, and to reach the general population of Paraguay. The bias of the educational level could be attributed to a “self-selection” of people interested in joining the study and their level of access to social networks: considering the increased availability of internet for people with higher levels of education.

In conclusion, the general score of perceived stress during the quarantine period was 18.10 ± 5.99 (moderate self-perceived stress level). Significant associations were found between stress, female sex, and being single. It may be also understandable that social restrictions may worse loneliness among single subjects.

Considering the findings of this study, we suggest mental health services to provide a phone-based or web-based psychological support to the general population, when required by the general practitioner, and additional regular phone-based or web-based follow-up to all their users to contrast the effect of pandemic on preexisting mental disorders and prevent relapses or suicide (30, 31). This approach may improve the accessibility to mental healthcare services in Paraguay, especially in times of social distancing. It is of note that this may only be accomplished by an effort by public and/or private organizations in order to provide necessary equipment and resources to reach all levels of general population.

## Data Availability Statement

The original contributions presented in the study are included in the article/supplementary material, further inquiries can be directed to the corresponding author/s.

## Ethics Statement

The studies involving human participants were reviewed and approved by Ethics Committee, National University of Caaguazú. The patients/participants provided their written informed consent to participate in this study.

## Author Contributions

JT, CR-G, IB, MO'H, OG, and AV conceptualized the structure and design of the manuscript. JT, MO'H, IB, OG, IG, and JC-M wrote the first draft of the manuscript. CR-G and IB designed the first draft of the survey questionnaire. IB, JT, IG, JC-M, and AV analyzed the data. All authors provided critical intellectual contribution to the manuscript, reviewed, and approved the final version of the manuscript.

## Conflict of Interest

The authors declare that the research was conducted in the absence of any commercial or financial relationships that could be construed as a potential conflict of interest.
